# 

**Published:** 1858-03

**Authors:** 


NORTH AMERICAN

### MEDICO-CHIRURGICAL REVIEW.

Vol. II.] MARCH, 1858. [No. 2.

### CONTENTS.

### ANALYTICAL AND CRITICAL REVIEWS.

ART. PAGE

I.—A Practical Treatise on Diseases of Children. By J. FORSYTH MEIGS, M.D. . . . . . . . . . . . . 209

II.—1. Researches in Physiology and Pathology. By E. BROWN-SÉQUARD, M.D. . . . . . . . . . . . . 222

2. Researches on Epilepsy. By the same author . . . 222

3. Essays on the Secretory and Excito-Secretory System of Nerves. By HENRY F. CAMPBELL, M.D. . . . . . . . 222

III.—Diseases of the Alimentary Canal. By S. O. HABERSHON, M.D., etc. 236

IV.—Treatise on Midwifery, etc. By P. CAZEAUX . . . . . 252

V.—Human Histology. By E. R. PEASLEE, M.D. . . . . . 260

VI.—Pathological Chemistry. By MM. BECQUEREL and RODIER . 263

VII.—Manual of Detection of Poisons. By DR. F. JUL. OTTO, M.D. . . 264

VIII.—Botanical Text-Book. By ASA GRAY, M.D., etc. . . . . 265

### ORIGINAL COMMUNICATIONS.

I.—Prominence of the Eyeball, etc., after the Ordinary Operation for Strabismus; with description of a New Pair of Scissors for Performing the Sub-conjunctival Operation. By ADDINELL HEWSON, M.D., etc. . . . . . . . . . . 266

II.—On the Injection of Urea and Other Substances into the Blood. By W. A. HAMMOND, M.D. . . . . . . . . 287

III.—Epidemic Dysentery By W. J. JOHNSON, M.D. . . . . . 293

IV.—Medullary Sarcoma of the Liver. By JOHN P. KLUGE, M.D. etc. 307

V.—Compound Comminuted Fracture of the Humerus. By J. MECKLEY, M.D. . . . . . . . . . . . . . . 310

VI.—Pueperal Convulsions from Gastric Irritation. By J. LEVERGOOD, M.D. . . . . . . . . . . . . . . . 311

VII.—Proceedings of the Pathological Society of Philadelphia . . 312

VIII.—Mortality Statistics of the U.S. Census for 1850. By E. READ, M.D. . . . . . . . . . . . . . . . 334

ART. PAGE

AMERICAN PROGRESS OF MEDICAL SCIENCE.

Report on Practical Medicine. By AUSTIN FLINT, M.D., Professor of Pathology and Clinical Medicine, University of Buffalo . . 341

BI-MONTHLY PERISCOPE.

PHYSIOLOGY.

1. Functions of the Right Auricle . . . . . . . . . 378
2. Action of the Pancreatic Juice and Bile . . . . . . . 379

SURGERY.

1. Collodion in Orchitis . . . . . . . . . . . . 379
2. Treatment of Primary Syphilis . . . . . . . . . . 380
3. Syphilitic Inflammation of the Retina . . . . . . . . 380
4. Diagnosis of Hydrocele . . . . . . . . . . . 381
5. Reduction of Hernia by Compression . . . . . . . 382
6. New Treatment for Acute Glaucoma . . . . . . . . 382

PATHOLOGY AND MEDICINE.

1. Negroes not exempt from Southern Epidemics . . . . 383
2. Clinical Observations on Epilepsy . . . . . . . 384

THERAPEUTICS.

1. Antidote to the Poison of the Rattlesnake . . . . . . 385
2. Therapeutic Action of, and New Mode of Administering Chlorate of Potash . . . . . . . . . . . . . . . 387

EDITORS' TABLE.—Suits for Malpractice . . . . . . . . 389
African Edibles . . . . . . . . . . . . 391
Thermal Springs . . . . . . . . . . . . 394
Monstrosity . . . . . . . . . . . . . 397
New Medical Journal . . . . . . . . . . . 397
Fusion . . . . . . . . . . . . . . 398
Prizes at the Académie de Médicine . . . . . . . 398
Editorial Change . . . . . . . . . . . . 398
Animal Signatures . . . . . . . . . . . . 398
Natural Anæsthesia . . . . . . . . . . . . 399
Hippophagy . . . . . . . . . . . . . 399
Quarantine Convention . . . . . . . . . . . 399
Bi-Monthly Bibliographical Record . . . . . . . . 400

